# Exploring Dabai (*Canarium odontophyllum*), Indigenous Fruit of Borneo: A Review of Nutritional Values, Potential Uses, Emerging Application in Essential Oil Processing, and Health Benefits

**DOI:** 10.3390/plants11192646

**Published:** 2022-10-08

**Authors:** Muhammad Hazwan Hamzah, Mohd Salahuddin Mohd Basri, Bernard Maringgal, Maimunah Mohd Ali, Mohd Hafizz Wondi, Hasfalina Che Man, Sukardi Gatuk Abdulloh

**Affiliations:** 1 Department of Biological and Agricultural Engineering, Faculty of Engineering, Universiti Putra Malaysia, Serdang 43400, Selangor, Malaysia; 2 SMART Farming Technology Research Centre, Faculty of Engineering, Universiti Putra Malaysia, Serdang 43400, Selangor, Malaysia; 3 Department of Process and Food Engineering, Faculty of Engineering, Universiti Putra Malaysia, Serdang 43400, Selangor, Malaysia; 4 Laboratory of Halal Science Research, Halal Products Research Institute, Universiti Putra Malaysia, Serdang 43400, Selangor, Malaysia; 5 Laboratory of Biopolymer and Derivatives, Institute of Tropical Forestry and Forest Products, Universiti Putra Malaysia, Serdang 43400, Selangor, Malaysia; 6 Faculty of Resource Science and Technology, Universiti Malaysia Sarawak, Kota Samarahan 94300, Sarawak, Malaysia; 7 Faculty of Plantation and Agrotechnology, Universiti Teknologi MARA Cawangan Sarawak, Kampus Mukah, K.M 7.5 Jalan Oya, Mukah 96400, Sarawak, Malaysia; 8 Department of Agro-Industrial Technology, Faculty of Agricultural Technology, Universitas Brawijaya, Jl. Veteran, Malang 65145, Jawa Timur, Indonesia

**Keywords:** dabai, food products, health benefits, nutrition, indigenous fruit

## Abstract

Dabai (*Canarium odontophyllum*) is a fruit-bearing plant native to Borneo. Its fruit is an indigenous seasonal fruit that is considered to be underutilized due to its short shelf life. However, new products have been developed to ensure a continuous supply of dabai fruit throughout the year. Hence, the exploration of dabai fruits in characterizations and utilization for food products and essential oil has expanded exponentially. This review addresses the nutritional values, health benefits, potential food products, and essential oil processing of dabai fruit. All parts of dabai fruit, such as the pulp, skin, and kernel, contain a considerable amount of bioactive compounds, dietary fiber, and nutrients. Moreover, dabai fruit has also been proven to have health benefits such as an antioxidant capacity, cholesterol reduction, diabetes type 2 prevention, and reduction in the risk of heart disease. Some potential dabai-based food products and oil processing of dabai are also highlighted. The future perspectives and challenges concerning the potential uses of dabai are critically addressed at the end of this review. Based on this review, it is proven that dabai has various health benefits and represents a potential breakthrough in the agricultural and food industries.

## 1. Introduction

Dabai (*Canarium odontophyllum*) is a fruit-bearing plant native to Borneo from the Burseraceae family. Dabai fruit is an indigenous seasonal fruit considered a delicacy in Sarawak. The skin and flesh are hard and inedible when ripe, so the fruit needs to be soaked in warm water for up to 10 min to soften the skin. The taste of the flesh is similar to avocado due to its creamy texture and fatty taste. The kernel is edible and rich in oil yet normally discarded. Dabai is devoured as a snack food by locals, with high nutritional values that are good for health. Despite its abundance, the potential of dabai fruit has not been comprehensively explored and it is considered to be an underutilized fruit [1]. In addition, dabai fruit has a minimal shelf life. It can be stored at room temperature for only up to three days before the skin becomes crinkled due to moisture loss [2]. This property is becoming a significant drawback to the distribution and marketing of dabai fruit nationally and internationally. To expedite the growth of the dabai market, the Malaysian Agricultural Research and Development Institute has developed dabai pickles and frozen dabai pulp to ensure a continuous supply is available even during the off seasons [3]. The developed products enable steady processing of dabai fruit throughout the year and enhance its potential to be further explored.

Recently, the nutritional values and health benefits of dabai have becoming more recognized and well investigated. The emerging trend of studies reported in the literature is towards profiling the physico-chemical and biological contents of dabai skin, flesh or pulp, kernel, and leaf and exploring their potential health benefits. The degree of maturity, climate, and postharvest environments are a few elements that affect the physico-chemical and biological characteristics of fruits [4]. A saturated fatty acid oleoresin has been extracted from dabai pulp. It has been proven to contain low peroxide and free fatty acids and is rich in vitamin E (α-tocopherol) [5]. Previous research also evaluated the efficiency of dabai pulp oil (DPO) and defatted dabai pulp (DDP) in lowering hypocholesterolemic levels and providing hepatoprotective benefits in hypercholesterolemic rats [6]. DDP, rich in anthocyanin and syringic acid, significantly lowers cholesterol, low-density lipoprotein, and β-hydroxy β-methylglutaryl-CoA (HMG-CoA) reductase, a rate-controlling enzyme of a metabolic pathway that produces cholesterol. A study by Salleh et al. [7] revealed the excellent antioxidant capacities of edible dabai fruit components such as dabai kernel, skin, and pulp. Dabai kernel also has a high content of dietary fiber, up to 22%, which is good for lowering type 2 diabetes and the risk of heart disease [8].

Due to the versatility of dabai fruit, it can be transformed into various food products. Oleoresin, a natural plant product primarily containing essential oil and resin, is one of the potential products of dabai fruit. By utilizing supercritical fluid extraction, Abdul Kadir et al. [5] successfully removed all toxins from oleoresin, which was extracted from dabai pulp. The extracted oleoresin can be used as a new alternative fat to be incorporated in the formulation of end products in food industries. Azlan et al. [9] utilized dabai fruit components, oleoresin, oil, and kernel to make dabai cocoa bars. Dabai kernel fat partially replaced cocoa butter and oil in the cocoa bar formulation. Currently, there are various food products with dabai incorporated, such as dabai fried rice, dabai sauce, dabai mayonnaise, dabai ice cream, and dried dabai [2]. With the current food research and development technology, more products can be developed to utilize dabai fruitfully. By taking advantage of the complete utilization of dabai fruit, the potential for profitable goods in food industries and other sectors could provide a wide variety of food-based and waste-processing products with high economic importance. Thus, this review aims to provide a comprehensive overview of the nutritional values, health benefits, potential food products, and essential oil processing of dabai fruit. With these findings, food manufacturers and researchers may gain fundamental knowledge to explore new perspectives for dabai products.

## 2. Dabai Physiology and Characteristics

In addition to ‘dabai’, kembayau is the name given to the fruit in both Brunei and Sabah [10]. It can be found in abundance in the tropical forests of east Malaysia, particularly in the Sarawak regions of Sibu and Kapit [11]. It is a fruit that is only available during certain times of the year, and depending on the climate, the best times to harvest it are between July and August, and November and December. The plant can be grown from seeds on the island of Borneo in Southeast Asia [12].

Approximately 100 different species of *Canarium* L. can be found in the tropical forests of Africa, Asia, and Australia. However, the species of *Canarium odontophyllum* can only be discovered in the tropical forests of Sumatra (Indonesia), Borneo (Malaysia and Indonesia), and the Philippines, more specifically, on the islands of Mindanao and Luzon [13]. According to Leenhouts et al. [14], out of the 52 species recorded as belonging to the genus *Canarium* L., 8 of these species can be found on the Malay Peninsula and 14 of these species can be found in Borneo. The names of the species, such as *Canarium perlisanum* and *Canarium sumatranum*, are often coined from their origin. However, in some cases, the plant is also somehow indigenous to several other regions.

A young dabai tree can produce up to 10 kg of dabai, whereas a mature tree (10 years old or older) can produce between 80 and 100 kg [15]. Therefore, it was anticipated that around 200,000 to 500,000 kg would be produced throughout each season. The tree has the potential to grow between 30 and 40 m in height and has a lifespan of about 40 years [16]. After 5 years of planting, the plant will begin to produce fruit, and a fully mature tree can produce up to 800 kg of fruit all at once [17]. *Canarium* L. species can grow naturally in a wide range of soil types. The optimal growing environment for these plants is land that is moist, rich, deep, crumbly, organically sandy loam and has a pH range between 4.5 and 6.5. Moreover, they can survive under alkaline conditions up to a pH of 7.4 and are capable of being contained in poorly drained wooded environments [18,19].

**Figure 1:**
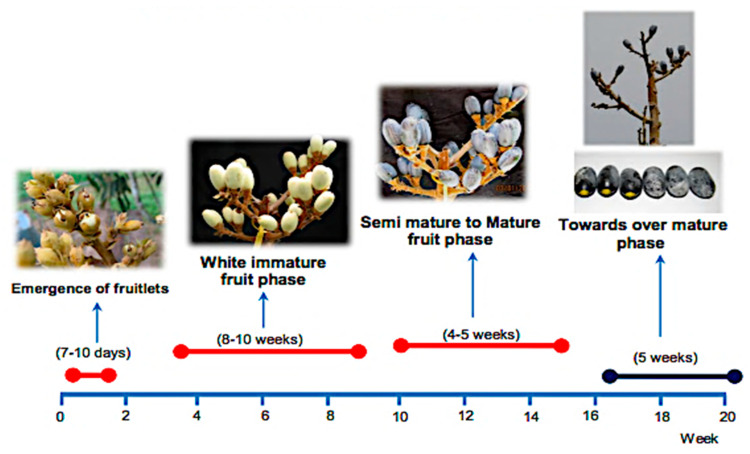
Timeline chart of dabai fruit during the maturation process. Adapted from [29].

The dabai fruit is physically characterized by its oblong shape, thin and edible skin, and white or yellow flesh, depending on the variety. It also has a peculiar flavor [20]. The color of the dabai fruit’s skin, when it is fully ripe, is somewhere between black and blue. Anthocyanin (cyanine-3-glucoside) is primarily responsible for the color of the pigment found in the skin [21,22]. The shape of the dabai fruit ranges from oval to ellipsoid, approximately 3.5 to 4.0 cm in length and 2.0 to 2.5 cm wide. The weight of a single dabai fruit can reach up to 18 g, and the structure of the fruit comprises pulp, skin, and kernel [23]. This observation was made previously by Prasad et al. [24], who indicated that it is oblong, measuring between 3 and 4 cm in length, and weighing 10 to 13 g each. The dabai fruits have a texture and look comparable to that of olives. When it is unripe, this fruit is a pale green, but it turns a deep purple or almost black when it reaches maturity. Its flesh is bright yellow, and the seed is triangular with three angles. Figure 1 shows the maturation process of dabai starting from the emergence of fruitlets until the over-mature phase.

**Figure 2:**
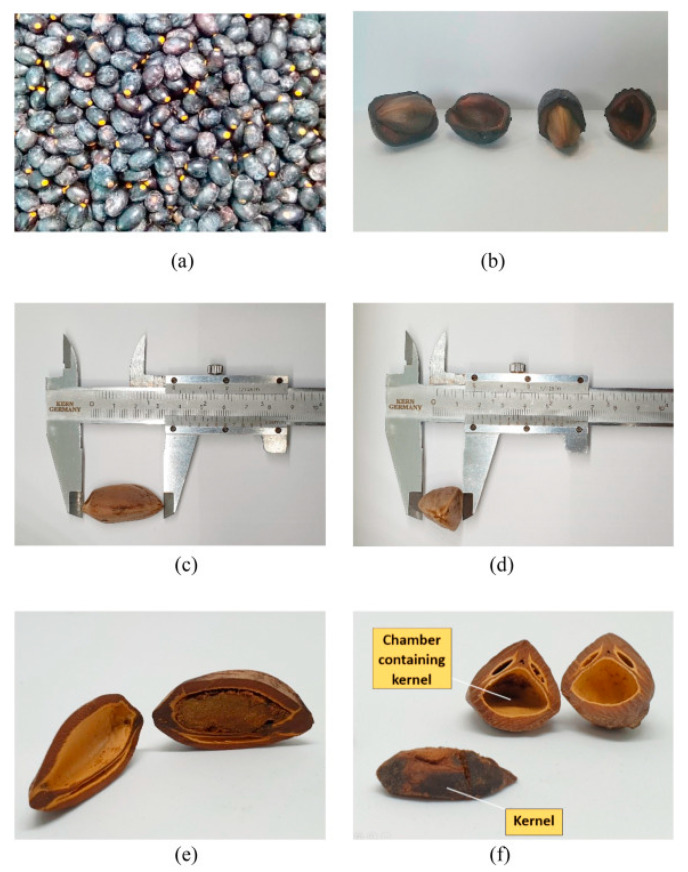
(**a**) Mature dabai fruits; (**b**) Dabai flesh (mesocarp) and hard seed (endocarp); (**c**) Length of the seed; (**d**) Width of the seed; (**e**) Longitudinal section of the seed; (**f**) Transverse section of the seed and the kernel. Adapted from [16].

The fruit is made up of an outer skin (epidermis) and a fleshy core (mesocarp) that surrounds a hard seed (endocarp) [25,26] Figure 2 shows parts of the dabai. The dabai seed has a tough and woody endocarp that encloses an edible cotyledon on the inside, and the physical features of this nutshell are similar to those of a palm kernel shell [11,27]. When mature, the fruits usually have a blue-black coloration and typically take on the appearance of plums or drupes. It is glabrous, particularly near the base and the tip, and has a rough texture. The pericarp can be fleshy or fibrous pyrene stony [28].

As shown in Figure 2a, Ideris et al. [16] also discovered that the mature skin of the fruit is black. Its immature state has a color range from white to a very light yellow. Figure 2b illustrates the seed and the flesh of the fresh fruit. The fruit has an ellipsoid or oblong shape, approximately 3 to 4 cm long and 2 to 2.5 cm wide. The flesh thickness is approximately 0.4 to 0.7 cm, covering a single large seed [10]. As shown in Figure 2c,d, the typical length of the seed is approximately 3 to 4 cm, and its width is about 1.5 to 2 cm. The seed has a cross-section in the shape of a sub-triangle, and it often contains three chambers [2]. The oil-rich kernel is found in the largest chamber, as shown in Figure 2e,f.

## 3. Nutritional Values of Dabai

Dabai consumption, marketing, and production have all increased significantly in the commercial market. This scenario has led to advancements and inspired researchers to take a closer look at the nutritional values of dabai. This section discusses the nutritional values, antioxidants, potential future alternative fat, and antimicrobial properties of dabai as one of the indigenous fruits of Borneo (Table 1).

### 3.1. Antioxidant and Nutritional Values

The high antioxidant properties and nutritional values make dabai appreciated by many people, especially the local community in Borneo. Consequently, there are compelling scientific grounds for investigating the phytochemical and nutritional composition of dabai. Dabai fruit has the most influential antioxidant properties. Faridah Hanim et al. [30] and Pin et al. [31] demonstrated that dabai peel is a major source of natural antioxidants. Prasad et al. [32] added that dabai peel and pulp extracts have a high concentration of carotenoids and a reasonable level of antioxidant capacity in the DPPH (2,2-diphenyl-1-picryl-hydrazyl-hydrate) and ABTS (2,2′-azino-bis(3-ethylbenzothiazoline-6-sulfonic acid)) assays. However, this depends on the geographical location of the growing dabai tree [34]. The fruit’s nutritional composition and antioxidant properties are influenced by the growing region or place of production, cultivar, climate, year of production, maturity, and cultural strategies. This is consistent with the work of Koczka et al. [47] and Arvaniti et al. [48], who showed that the antioxidant capacity of rosehip and figs was affected by the growing location.

The mean contractions of phytochemicals for their antioxidant benefit in maintaining longevity were studied by Basri et al. [49] to determine the inhibition of extracellular Ca^2+^ influx that is involved in extract-induced relaxation. After being incubated with EC_50_ of the extract, endothelium-denuded aortic rings were made to contract by phenylephrine (PE) and stimulated by potassium chloride (KCl). The control study did not involve preincubation with 30 min of extraction. Table 2 shows the effect of the CaCl_2_ content and reagent type on the contraction amplitude. Comparing the KCl- and PE-precontracted aortic rings to the control group, the vasocontraction responses to the cumulative concentration of Ca^2+^ did not exhibit any significant differences given the presence of dabai. The Ca^2+^-induced contraction that PE and KCl induced was not noticeably inhibited by dabai.

The total phenolics in dabai peels were found to be 18.88 to 26.62 mg gallic acid equivalent/g and the total flavonoid content was 15.62 to 46.70 mg quercetin equivalent/g [31]. However, this depends on the dabai peel extraction method. According to Khoo et al. [33], catechin and epigallocatechin were the most abundant phenolics in defatted dabai peel methanol extract. At the same time, ellagic acid was the most abundant phenolic acid in the water extract. Furthermore, the authors found that the defatted dabai peel contained more anthocyanidin than its pulp. High levels of total phenolics and Trolox-equivalent antioxidant capacity were found in the peel of a defatted dabai fruit extracted with methanol.

Calcium (16.00–67.88 mg/100 g fresh weight (FW)), sodium (7.26–11.19 mg/100 g FW), and potassium (3.64–7.19 mg/100 g FW) are the most abundant minerals in dabai fruit. Still, their concentrations vary significantly depending on the geographical location and variety [34]. In contrast, the study by Hoe and Kueh [35] indicated that the most potent minerals in dabai fruit are potassium (810 mg/100 g edible portion), calcium (200 mg/100 g edible portion), magnesium (106 mg/100 g edible portion), and phosphorus (65 mg/100 g edible portion). The authors also noted that dabai is high in energy (339 kcal/100 g edible portion) and protein (3.8%/100 g edible portion). Surprisingly, no vitamin C was found in dabai fruit. Chew et al. [34] reported that the dabai fruit protein was abundant in aspartic and glutamic acids, which comprised 45 to 49% of the total amino acids.

### 3.2. A Potential Future as Alternative Fat

Dabai has the potential to be a new source of alternative fat [50]. The most abundant fatty acids in the fruit are oleic (18:1), linoleic (18:2), and palmitic (16:0) acid, with percentages comparable to palm oil [37]. According to Umi Kalsum and Mirfat [36], the fruit has a low moisture content but high fat content. The fat content contributes to the high energy content in dabai fruit. The fruit contains 29.58 to 37.07% fat and contributes 504.12 to 567.12 kcal of energy in dabai fruit. These results are consistent with those of Hoe and Kueh [35], who reported that the fruit contained 26.2% fat and 339 kcal of energy.

Dabai pulp and kernel oils could be used to develop potentially healthy cooking oils due to their favorable fatty acid profile and high antioxidant activity [37]. These claims are consistent with those made by Basri et al. [20], who stated that dabai fruit is a good source of unsaturated fatty acids, and so has the potential to be converted into healthy cooking oil. On the other hand, dabai pulp and kernel fat has been shown in studies to boost the lipid profile of laboratory rabbits given dabai fat. In addition, in vitro and in vivo studies show that defatted dabai peel has a modest cardioprotective effect [38,39]. This may be due to the high dietary fiber and antioxidant activity in dabai fruit, which contributes to the cholesterol-lowering effect. This finding provides evidence that dabai defatted pulp might be able to lower cholesterol and protect cells from damage. A study by Khoo et al. [40] examined the potential use of defatted dabai peel in future bioceuticals. This study revealed that defatted dabai peel had the highest antioxidant capacity and an oxidative-stress-inhibiting effect. The defatted dabai peel increases cellular antioxidant enzymes, i.e., superoxide dismutase and glutathione peroxidase, in rabbits, and inhibits lipid peroxidation, i.e., plasma malondialdehyde.

Recently, Azlan et al. [9] found that the high saturated fat of dabai kernel can be used in place of cocoa butter or palm kernel fat to produce chocolate. Moreover, the authors highlighted that sensory analysis revealed that chocolate bars made from dabai kernel were preferred over dabai oil and oleoresin. This has significant potential for the chocolate bar industry. Similarly, Abdul Kadir et al. [41] indicated that dabai pulp oleoresin is safe to be consumed and has significant potential as a new margarine and cocoa butter substitute with bioactive compounds (vitamin E). The authors also mentioned that the low amount of peroxide values (5.60 ± 0.09 mEq/kg) and free fatty acids (3.40 ± 0.03%) in dabai pulp oleoresin shows that it is of good quality and can be used for more than just food.

### 3.3. Antimicrobial Properties

The antimicrobial properties of dabai fruit have been extensively researched. For instance, Basri et al. [42] found that *Candida glabrata* was susceptible to the dabai pulp extract. Next, Basri et al. [43] experimented with dabai seed and showed promising antibacterial activity against infections associated with *Acinetobacter baumannii* and *Proteus mirabilis*. The findings suggest that dabai fruit could be a valuable source of an alternative phytotherapeutic agent with antimicrobial potential. In another study, Basri et al. [44] found antimicrobial effects of stem bark extracts from dabai in methanol, acetone, and distilled water against *Staphylococcus aureus* ATCC 25923, *Bacillus cereus* ATCC 6633, *Escherichia coli* ATCC 25932, *Pseudomonas aeruginosa* ATCC 27853, *Acinetobacter baumannii* strain sensitive, *Candida albicans* ATCC 64677, *Candida glabrata* ATCC 90028, *Aspergillus niger*, and *Fusarium solani* M2781. They discovered that an extract of the stem bark of the dabai tree, when processed with methanol and acetone, had a bactericidal effect against *Staphylococcus aureus* and a bacteriostatic effect against *Acinetobacter baumannii*.

Research into dabai leaves has been started and found to have significant potential in the future pharmaceutical and nutritional food-based industries [45,51]. The potential of leaf extracts of dabai as an antimalarial agent has been extensively investigated by Ishak et al. [46]. This study found that dabai leaf extract in methanol may inhibit plasmodium at 5% parasitemia at different morphological stages, such as young trophozoite, mature trophozoite, and schizont. This study indicated that the leaf extract of dabai can be further developed into an antimalarial drug. A seminal study in this area is the work of Shamsuddin et al. [45]. They revealed that combining dabai leaf extract with acetone could provide insight into the antimicrobial mechanism, potentially leading to the identification of a target protein for future novel therapeutic development against methicillin-resistant *Staphylococcus aureus* infections.

## 4. Potential of Food Products and Essential Oil Processing of Dabai

The food industry is interested in incorporating dabai into various food products due to its high nutritional value and abundant chemical composition. Dabai production and consumption also result in significant amounts of solid waste, which raises the possibility of valuable waste-processing products. Only 43 to 56% of each dabai fruit is pulp; the remainder, which includes the seed (6–9%) and peel (7–16%), can be considered wastes [8]. The potential food-based and waste-processing products related to dabai are summarized in Table 3.

After peeling the pulp and removing the seed, a substantial amount of dabai pulp is typically used in numerous food-based products. Dabai has a delicious creamy taste and a pleasant aroma. Dabai fruit is a good source of high energy, fat, protein, and minerals such as magnesium, phosphorus, and calcium [8]. In the food industry, the addition of dabai to a cake batter can improve the quality of the cake produced due to its nutrients [58]. A cake containing 10 g of dabai was the most favored in terms of general acceptability, with a mean score of 6.90. Ing [60] developed a formulation of a mixed fruit juice from dabai fruits and watermelon. The best mixture of 37% of total fruit puree with a 1:1 watermelon to dabai fruit ratio, 8% sugar, 0.1% pectinase enzyme, 0.1% citric acid, 0.05 sodium benzoate, and 54.75% water had a shelf life of 3 weeks. A paste made from the creamy fresh or preserved flesh of the dabai fruit is the main component of a savory stir-fried rice with dabai [61].

Furthermore, the commercial market offers a variety of processed dabai products or ingredients, such as mayonnaise, ice cream, dried fruit, crackers, pickle, sauce, cocoa bars, and peanut spread [9,23,57]. Dabai extract or oil contains antioxidants, including phenolics, flavonoids, anthocyanidins, and carotenoids, which protect the skin from sun damage and slow the ageing process [50,55]. The fatty acid profile of dabai pulp oil is similar to that of palm oil. As a result, dabai pulp oil can most likely be used as a vegetable oil substitute [62]. Growing recognition of the value of vegetable oils and their health benefits in achieving sustainable development goals has sparked economic interest in their ability to be transformed into agricultural and industrial products.

In the preceding context, an area of research that has gained significant interest is the processing of dabai oil. The process configuration includes the extraction time, solvent, temperature, and other factors that can modify the physico-chemical properties of dabai oil and the morphology of dabai samples. Table 4 summarizes the dabai oil extraction method and the parameters involved. Dabai pulp and kernel oils have been extracted using supercritical carbon dioxide (CO_2_) for their antioxidant and fatty acid properties, their potential as a new alternative fat, and their potential to enhance the treatment of hypercholesterolemia [5,6,25,55,56]. Other fields, such as solvent extraction for oil composition analysis, supplementation to normocholesterolemic and hypercholesterolemic patients, hepatotoxicity, and protective activities in an in vivo model, are also being investigated [8,12,13,37,39]. However, supercritical extraction has drawbacks such as high investment costs and difficulties with polar analyte extraction due to its low polarity [63]. In addition, the toxicity of the solvent used and process selectivity must be considered during the manufacturing process [6]. There is a constant need to search for an optimal technology for the production process while considering its cost, available materials, and efficiency. A fruitful area for further work is an exploration of the promising green environmentally methods for oil extraction from dabai fruit.

## 5. Health Benefits

**Figure 3:**
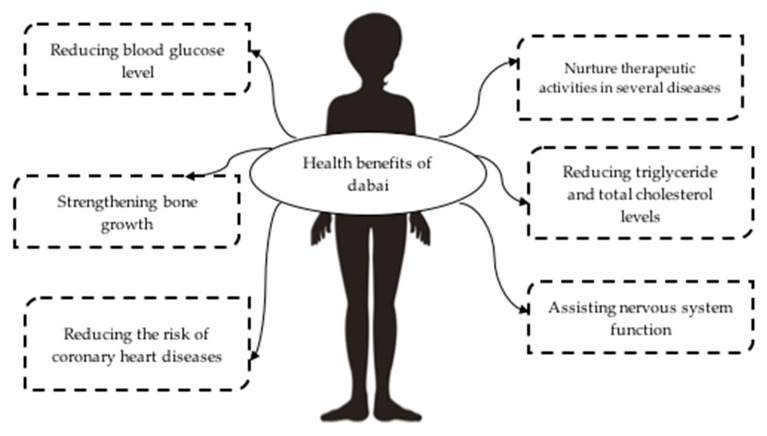
Potential health benefits of dabai.

Dabai is abundant in nutrients and minerals that help in boosting human health. Numerous health benefits have been investigated in the pulp and kernel of dabai fruit, which could be explored in future research in terms of preservation and health enhancement through optimal storage conditions and processing technologies. In addition to their nutrient contents, the health aspects of dabai fruit are of great importance since various health issues such as skin problems, coronary heart disease, and cardiovascular diseases could be monitored by adjusting dietary ingestion of lipids and phenolic compounds. Although the fruit is very delicate and perishable, it is well known to have significant potential for health benefits. The potential health benefits of dabai are illustrated in Figure 3.

### 5.1. Bioactive Compounds

Dabai is rich in monomeric anthocyanin contents and flavonoids, which are essential for daily intake. In addition, the phenolic compounds found in dabai fruit could act as antioxidants and assist in preventing cancer. As a potential source of plant oil, dabai is a good source of dietary lipid since it could provide essential dietary antioxidants and contains low saturated fat levels. Dabai pulp oil is suitable for use in addition to vegetable oil since it contains a comparable fatty acid profile to palm oil, including oleic, palmitic, and linoleic acids [55]. It is also believed that the polyphenols in dabai fruit are responsible for improving lipid profiles.

### 5.2. Vitamins and Minerals

Dabai fruit has a high energy source, protein, fat, and essential minerals, including calcium, magnesium, and phosphorus [9]. Dabai is favored for use as a healthy oil and in the production of functional foods due to its fatty acid composition, which contains more than 50% monounsaturated and polyunsaturated fatty acids [8]. In addition to being nutritious, dabai is known for its high content of vitamin E, especially γ-tocopherol and α-tocopherol, typically found in the human body [37]. Vitamin E is a good antioxidant and important in health-promoting properties. These forms of vitamin E act as a radical chain-breaking antioxidant, which is vital for lowering the risk of cardiovascular disease and platelet aggregation. Hence, the multitude of nutrient compounds found in dabai are recommended for promoting the health benefits of the fruit to help in developing promising functionalities and commercial food products. In a study performed byDing and Tee [10], it was found that dabai fruit has a high energy content, fat, carbohydrate, crude fiber, protein, ash, and essential minerals such as calcium, phosphorus, potassium, magnesium, and iron. Calcium and magnesium in dabai are essential trace elements that are beneficial in strengthening bone growth and reducing the risk of coronary heart diseases [50].

### 5.3. Antioxidant Capacity

Chew et al. [34] successfully investigated the potential antioxidant properties of red and purple dabai fruits. It was revealed that the dabai fruit yielded the highest antioxidant capacity of 0.68 ± 0.09 mmol Trolox equivalent/g dry weight. The high antioxidant level in dabai could significantly delay the oxidation process of oxidizable substrates and nurture therapeutic activities in several diseases, including cancer, liver problem, and cardiovascular disease [56]. Faridah Hanim et al. [30] described the beneficial effects of dabai peel as the main source of antioxidants that could prevent lipid peroxidation and increase cellular antioxidant enzymes. The use of dabai has been shown to effectively increase lipoprotein lipase and antioxidant capacities, including catalase, glutathione peroxidase, and superoxide dismutase [56].

### 5.4. Anti-Cholinesterase Activity

Dabai also contains acetylcholinesterase, a key enzyme in the breakdown of acetylcholine. Acetylcholinesterase helps support the nervous system in battling the loss of cholinergic neurons caused by diseases such as Alzheimer’s disease [64]. The extraction from dabai has been proven to lower plasma cholesterol, which can reduce the amount of low-density lipoprotein, which can lead to atherosclerosis in the arteries [2]. In another study, Abdul Aziz et al. [1] revealed that dabai leaf extract could reduce the blood glucose level. Saponins, terpenoids, and flavonoids found in dabai extract could have a hypoglycemic effect to regulate pancreatic tissue for insulin production and monitor glucose absorption.

### 5.5. Cholesterol-Lowering Activity

A healthier diet plays a vital role in controlling cholesterol levels in the human body. Dabai has shown anti-obesity and cholesterol-lowering effects depending on the type of cultivars. The extraction of dabai pulp has been proven to be successful in lowering plasma cholesterol [2]. It is also noted that the functionality of dabai pulp oil is promising as it has an anti-hyperglycemic effect by reducing blood glucose levels. Apart from this, Azlan et al. [65] demonstrated that dabai pulp oil is essential in reducing triglyceride and total cholesterol levels.

## 6. Future Perspective and Challenges

One of Sarawak’s native fruits, the dabai, was recently determined to have significant potential for commercial exploitation. Dabai is a highly perishable crop, and few organizations have started shelf life studies on the preservation or minimization of quality loss [61,66,67]. The marketing life of dabai has been limited by its short shelf life. This also reduces the market potential of the fruit, particularly in terms of its ability to reach domestic and international markets. The fruit is very palatable, especially to the local populace, but inappropriate postharvest handling activities due to bacteria, insects, and respiration cause significant losses [68]. Fruit may be stored during the peak harvesting season and sold during the off-season when a widely employed process of freezing fruit is used. More broadly, there is still a need for long-term freshness and quality preservation research. Another important practical implication of the various technologies introduced is sensory evaluation. Sensory attributes such as flavor, texture, appearance, and taste are usually examined to evaluate the acceptable and quality interpretation of foods among consumers [69].

The National Agro-Food Policy 2.0 (DAN 2.0) 2021–2030, which the government recently introduced, enables the agro-food sector to be more competitive by applying highly agricultural technology, which is able to contribute to national economic development and ensure environmental sustainability in accordance with the 2030 Sustainable Development Goals [70]. Postharvest handling is more controlled and effective with emerging technologies, such as modified atmosphere packaging, bio-based coating or film materials, and smart packaging integrated with the Internet of Things, which could be improved further to extend the shelf life of fruits [66,71,72]. Other examples, such as rapid information on the structure, mechanical, physical, and chemical properties, could be provided by non-destructive testing without the use of techniques that negatively impact the aspect and quality of the food. However, this method suffers from the knowledge gap between laboratory applications and the field scale [73]. The digitization of production-based industries, including agro-food, is driven by technology drivers, including artificial intelligence, system integration, big data analytics, and advanced materials [74]. This is modernizing agriculture based on the 12th Malaysia Plan to spur economic growth with the development of the Fourth Industrial Revolution.

Recognizing the economic prospects of dabai fruit, the strategies outlined in DAN 2.0 include stepping up efforts to advance research and development, technology adoption and automation, supportive ecosystems, and various innovation programs and activities [70]. In order to produce dabai fruits with premium qualities and a long storage life for shipment to far-off markets locally and internationally, product research and development must be prioritized, and production techniques must be developed [75]. However, functional food product development provides opportunities and challenges for food manufacturers. The synergies of cooperation need to be enhanced, and leapfrog approaches based on the quintuple helix model, a combination of academia, universities, industry, communities, government, and the environment, utilized in providing solutions to the development of dabai-based food products [76]. Roundtable discussions, technology development collaborations, research papers, policy development, technology transfer, and training of the community need to be continued to ensure that the technology developed can be used effectively by the target group.

## 7. Conclusions

Dabai industries will provide various opportunities for economic investment, increasing the number of lower-income farmers and developing small and medium industries. Dabai is expected to contribute to the country’s gross domestic product growth because it has great potential as a source of genetic diversity of exotic fruits, nutritional values, and health benefits in community forests. A fast and reliable equipment for oil extraction that could be carried out by unskilled personnel on-site and for long hours of extraction activities would significantly facilitate the high-yield production of dabai oil. Such equipment would benefit oil producers or manufacturers and guarantee that the extracted oil meets standards for export. In addition to applications in food-based products, considerably more work will need to be carried out to determine the potential of dabai fruit for non-food-based products. Research on the genetic diversity is required as the foundation for breeding programs that may provide solutions to many issues that prevent this exotic fruit tree from becoming a crop. Vegetative propagation methods should be developed once a suitable clone has been found in order to turn it into a crop. A more thorough investigation could evaluate the impact of postharvest, handling and storage, quality and shelf life, and innovation on dabai fruit, particularly during the off-season. Therefore, it is anticipated that the commercialization of this indigenous fruit will increase significantly in the near future with various stakeholders’ contributions. A key policy priority should consequently be to plan for long-term care to move towards sustainable indigenous fruit management.

## Figures and Tables

**Table 1:** Nutritional values of dabai.

Nutritional Values	Dabai Part	Findings	References
Antioxidant and Nutritional Values	Peel	Major source of natural antioxidants	[30,31]
	Peel and Pulp	High concentration of carotenoids	[32]
		Contains total phenolic content, i.e., gallic acid, catechin, epigallocatechin, ellagic acid, and flavonoid	[31,33]
	Pulp	Contains minerals, i.e., calcium, sodium, potassium, magnesium, and phosphorus	[34,35]
		High in energy, protein, and total amino acids, i.e., aspartic and glutamic acids	[34,35,36]
Alternative Fat	Pulp	Low in moisture content but high in fat	[35,36]
		The most abundant fatty acids are oleic, linoleic, and palmitic	[37]
		Good source of unsaturated fatty acids	[20]
	Pulp and Kernel	Able to boost the lipid profile	[38,39,40]
	Pulp	Able to lower cholesterol and protect cells from damage	[38,39,40]
		Contains low amount of peroxide values and free fatty acids	[41]
Antimicrobial Properties	Pulp	Effective against *Candida glabrata*	[42]
	Seed	Promising antibacterial against infections associated with *Acinetobacter baumannii* and *Proteus mirabilis*	[43]
	Stem Bark	Antimicrobial effect on *Staphylococcus aureus* ATCC 25923, *Bacillus cereus* ATCC 6633, *Escherichia coli* ATCC 25932, *Pseudomonas aeruginosa* ATCC 27853, *Acinetobacter baumannii* strain sensitive, *Candida albicans* ATCC 64677, *Candida glabrata* ATCC 90028, *Aspergillus niger*, and *Fusarium solani* M2781	[44]
	Leaf	Potentially leading to the identification of a target protein for future novel therapeutic development against methicillin-resistant *Staphylococcus aureus* infections.	[45]
		Antimalarial agent	[46]

**Table 2:** Effect of the CaCl_2_ content and type of reagent on the amplitude of contraction.

Reagent	CaCl_2_ Content (mM)	Amplitude of Contraction (%) for Control	Amplitude of Contraction (%) by the Presence of Dabai
Phenylephrine (PE)	0.3	0	0
	1	26.9	61.9
	5	60.3	60.3
	10	100	100
Potassium chloride (KCl)	0.3	0	0
	1	73.0	31.7
	5	88.9	100
	10	100	31.7

**Table 3:** The potential food-based and waste-processing products related to dabai.

Dabai Part	Potential Uses	References
Pulp	Ice cream	[52]
	Peanut spread	[23]
	Sauce	[53]
Seed	Nut	[54]
	Biocarbon	[27]
Pulp and seed	Cocoa bar	[9]
	Essential oil	[12,38,55,56]
Pulp and peel	Juice, mayonnaise, dried fruit, crackers, pickle, sauce	[57]
	Fermented dabai	
	Cake	[58]
	Cookies	[59]
Pulp, peel and seed	Dried dabai	[20]

**Table 4:** Extraction method and parameters for essential dabai oil.

Extraction Method	Parameters	References
Ultrasound-assisted solvent extraction	Sample condition: Seed	[16]
	Amount of sample: 5 g	
	Amount of solvent: n-hexane to kernel powder: 50:1 in mL/g	
	Solvent: n-hexane	
	Extraction time: 45.79 min	
	Ultrasound amplitude level: 38.30%	
Solvent extraction	Sample condition: Dried pulp and seed	[37]
	Amount of sample: 2 g	
	Amount of solvent: N.S.	
	Solvent: Petroleum ether	
	Extraction time: 4 h	
	Sample condition: Dried pulp	[38]
	Amount of sample: 100 g	
	Amount of solvent: Chloroform:methanol (2:1, *v*/*v*) at a ratio of 1:5 (*w*/*v*)	
	Solvent: Chloroform, methanol	
	Extraction time: N.S.	
	Sample condition: Dried pulp or seed	[12]
	Amount of sample: 100 g	
	Amount of solvent: Chloroform:methanol (2:1, *v*/*v*)	
	Solvent: Chloroform, methanol	
	Extraction time: N.S.	
	Sample condition: Dried pulp, peel, and seed	[8]
	Amount of sample: 10 g (homogenized)	
	Amount of solvent: 180 mL	
	Solvent: Petroleum ether	
	Extraction time: 10 h	
	Sample condition: Dried pulp	[13]
	Amount of sample: 100 g	
	Amount of solvent: Chloroform-methanol-water (2:2:1.8 *v*/*v*) mixture	
	Solvent: Chloroform-methanol-water	
	Extraction time: 12 h	
	Sample condition: Dried skin, pulp, and seed	[7]
	Amount of sample: 100 g for each fraction	
	Amount of solvent: N.S.	
	Solvent: n-hexane	
	Extraction time: N.S.	
	Sample condition: Dried pulp (powder was no less than 0.2 mm in diameter)	[25,41]
	Amount of sample: 62.46 kg	
	Extraction time: N.S.	
	Pressure: 40 MPa	
	Temperature: 40 °C	
	CO_2_ flow rate: N.S.	
Supercritical CO_2_ extraction	Sample condition: Dried pulp and peel	[55]
	Amount of sample: 8 g	
	Extraction time: 30–60 min	
	Pressure: 20–50 MPa	
	Temperature: 40–60 °C	
	CO_2_ flow rate: 2 mL/min	
	Sample condition: Dried pulp	[56]
	Amount of sample: 48.92 kg	
	Extraction time: N.S.	
	Pressure: 40 MPa	
	Temperature: 40 °C	
	CO_2_ flow rate: N.S.	
	Sample condition: Dried pulp (powder was no less than 0.2 mm in diameter)	[6]
	Amount of sample: N.S.	
	Extraction time: N.S.	
	Pressure: 40 MPa	
	Temperature: 40 °C	
	CO_2_ flow rate: N.S.	
	Sample condition: Dried pulp (powder was no less than 0.2 mm in diameter)	[5,9]
	Amount of sample: N.S.	
	Extraction time: 2 h	
	Pressure: 40 MPa	
	Temperature: 40 °C	
	CO_2_ flow rate: 15 g/min, 30 min static duration	

Note: N.S.—not stated.

## Data Availability

Not applicable.
